# Differentiation of Hispanic biogeographic ancestry with 80 ancestry informative markers

**DOI:** 10.1038/s41598-020-64245-4

**Published:** 2020-05-08

**Authors:** Casandra H. Setser, John V. Planz, Robert C. Barber, Nicole R. Phillips, Ranajit Chakraborty, Deanna S. Cross

**Affiliations:** 1 University of North Texas Health Science Center; Department of Microbiology, Immunology, and Genetics, Fort Worth, TX USA; 2 University of North Texas Health Science Center; Department of Physician Assistant Studies, Fort Worth, TX USA

**Keywords:** Computational biology and bioinformatics, Evolutionary genetics, Genetic markers

## Abstract

Ancestry informative single nucleotide polymorphisms (SNPs) can identify biogeographic ancestry (BGA); however, population substructure and relatively recent admixture can make differentiation difficult in heterogeneous Hispanic populations. Utilizing unrelated individuals from the Genomic Origins and Admixture in Latinos dataset (GOAL, n = 160), we designed an 80 SNP panel (Setser80) that accurately depicts BGA through STRUCTURE and PCA. We compared our Setser80 to the Seldin and Kidd panels via resampling simulations, which models data based on allele frequencies. We incorporated Admixed American 1000 Genomes populations (1000 G, n = 347), into a combined populations dataset to determine robustness. Using multinomial logistic regression (MLR), we compared the 3 panels on the combined dataset and found overall MLR classification accuracies: 93.2% Setser80, 87.9% Seldin panel, 71.4% Kidd panel. Naïve Bayesian classification had similar results on the combined dataset: 91.5% Setser80, 84.7% Seldin panel, 71.1% Kidd panel. Although Peru and Mexico were absent from panel design, we achieved high classification accuracy on the combined populations for Peru (MLR = 100%, naïve Bayes = 98%), and Mexico (MLR = 90%, naïve Bayes = 83.4%) as evidence of the portability of the Setser80. Our results indicate the Setser80 SNP panel can reliably classify BGA for individuals of presumed Hispanic origin.

## Introduction

It is important to study the genetics of Hispanic populations to avoid oversimplifying this heterogeneous ethnicity into a single conglomerate. The identification of specific biogeographic ancestries (BGA) has implications both in clinical^1^ and forensic^2^ genetics. Clinically, a more complete description of the various Hispanic BGAs may result in identification of rare variants that may not have been previously described when grouping all Hispanic populations together^3^, or for controlling for population substructure in clinical trials^4,5^. Hispanic individuals are known to have differential predispositions for various diseases and ignoring this diversity restricts the generalizability of the results^6^. In forensics, BGA data could be used to investigate the origin of unidentified human remains (UHR)^7^, or locate the rightful parents/guardians of a child who is unable to identify where she/he is from^8^. It is the heterogeneous nature of Hispanic populations that has previously deterred full characterization of their substructure. However, in the past decade, there has been a movement to explore global human diversity and a variety of genetic panels have been designed for this purpose.

Early ancestry informative marker (AIMs) panels are “continental” in nature, focused on admixture mapping to determine from which of the six inhabited continents an individual has ancestry; these include: Seldin128^9^, Galanter *et al*.’s 446^10^, Kidd55^11^, EUROFORGEN^12^, Genetic Atlas^13^, Genographic Project^14^, Cuba by Marcheco-Teruel *et al*.^15^, and Cuba by Fortes-Lima *et al*.^16^. Although these studies assessed continental ancestry proportions (e.g. Seldin128)^9^, highly differentiated populations may be detected within continental panels, even identifying admixed populations such as Gujarati Indians in Houston, TX and Mexican ancestry from Los Angeles, CA^17^. The ability to separate small admixed populations among larger more homogenous populations supports the notion that continental SNPs with high genetic differentiation may still be informative on a more specific country level. The simultaneous description of highly divergent populations alongside less specific populations using the same SNP panel is central to the goals of our study. However, dual level analysis of admixed populations within continental panels is rare, as it tends to decrease the panel’s performance^2,17^.

Other panels target more specific, country BGA beginning in European populations before extending to other regions of the world (e.g. Denmark within Northern Europe). Although the Genographic Project^14^ assessed populations worldwide (though sparsely in the Americas), their in-house geographic population structure (GPS) algorithm is capable of identifying country of origin. EASTASAIMS was one of the first non-European AIMs panels focusing on 22 East Asian populations using 1,500 AIMs and was able to separate the five largest populations in the region^18^. Zeng *et al*.^19^ created a panel of 23 AIMs using F_ST_ focusing on the four major US populations from HapMap 3^20^: African ancestry from Southwest United States (ASW), Utah residents with Northern and Western European ancestry (CEU), Chinese from Metropolitan Denver, Colorado (CHD), and Mexican ancestry from Los Angeles, CA (MEX). And more recently, Huerta-Chagoya *et al*.^21^ reported 32 AIMs within Mexican mestizo populations, to estimate admixture proportions in various regions of Mexico.

Highly accurate BGA predictions are possible with up to 83% accuracy, but at the expense of panel size, requiring 40,000–130,000 SNPs as used in the Genographic Project^14^. Additionally, of the 12,476 reference samples used to select 40,000+ SNPs in their panel, only 9% were from American/Amerindian populations^22^, which limits the utility of their panel for resolving Hispanic ancestry. The size of this panel^14^, the proprietary nature of the SNPs on their Genochip^22^, and poor representation of the Western hemisphere, has prompted us to create a small, efficient, and publicly available SNP panel concentrated on BGA of Central America, South America, and the Caribbean.

Within one country, both Great Britain^23^ and Cuba^15,16^ have attempted to describe the diversity of their populations. The British Isles were ideal candidates for national differentiation due to their relative homogeneity and the presence of a geographic barrier which has historically restricted continuous gene flow with continental Europe and other island populations. In contrast, studies by Marcheco-Teruel *et al*.^15^, and Fortes-Lima *et al*.^16^ superficially appear to differentiate between the fifteen Cuban provinces on a national level, but their real focus was measuring admixture proportions using a subset of Galanter *et al*.’s 446 SNPs^10^, making their studies better described as continental and highlighting the need for a within country panel. Overall, at least 21 AIMs panels have been reported; however, of the 1,397 SNPs identified by Soundararajan *et al*.^24^, only 46 Consensus SNPs were in common to three or more SNP panels.

At present, there is no AIMs panel that focuses on the determination of BGA between countries in the Americas. Despite the overlap of our region of interest with the Galanter *et al*.’s 446 Latin American AIMs^10^, our purpose was to classify BGA, not to estimate the ancestral proportions contributed from 3–4 continental populations. The majority of AIMs panels and genetic ancestry studies have a heavy concentration of populations in Europe and Asia and far fewer in Central America, South America, and the Caribbean^13,14,18^. Our country panel addresses this gap in knowledge and focuses on these same populations.

## Results

### Setser80 SNP panel evaluation

**Figure 1:**
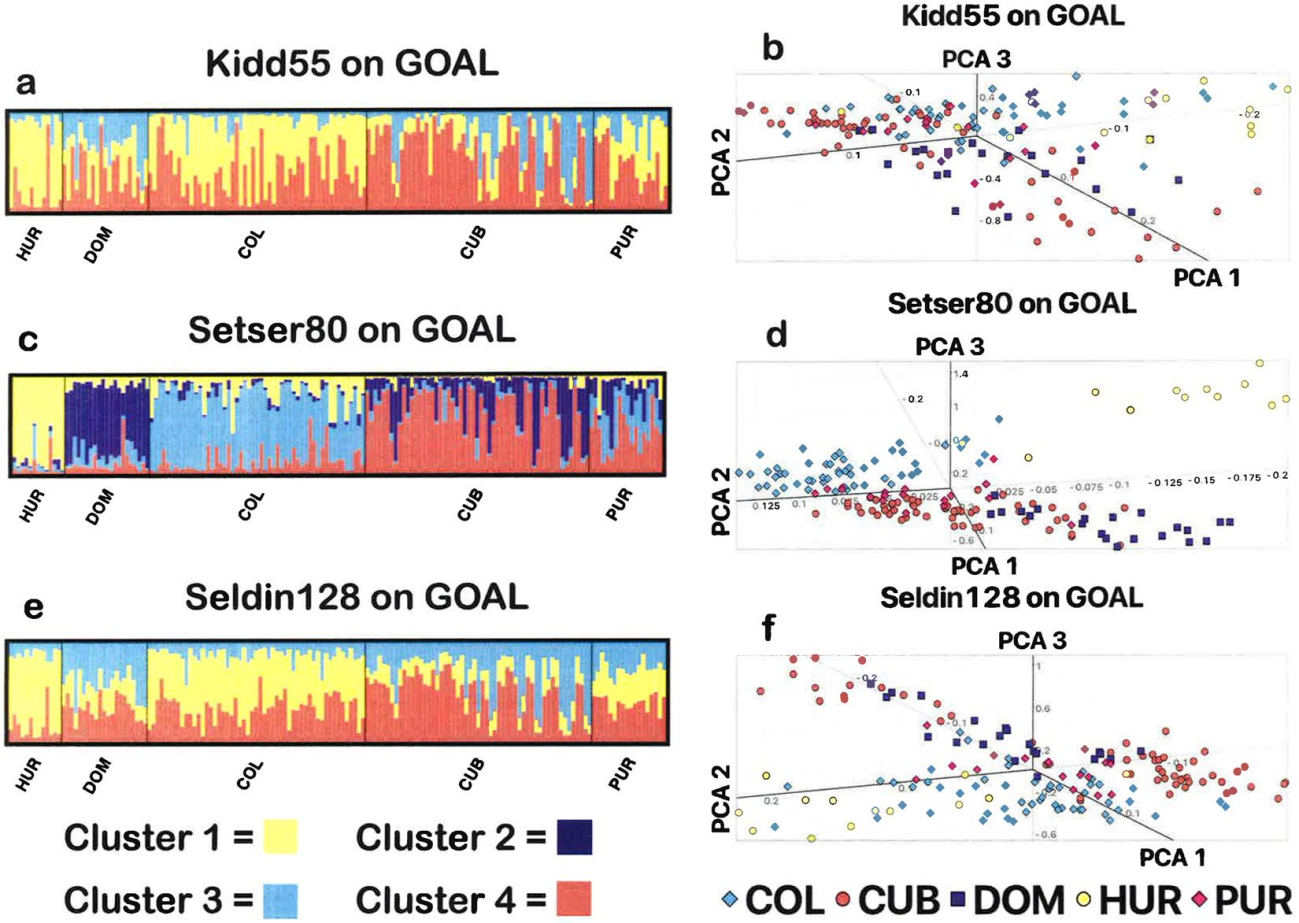
Comparison to other panels. Each plot represents 160 unrelated GOAL individuals and their respective populations. Figures a, c, and e are STRUCTURE plots where each vertical line represents one person. Figures b, d, and f are PCA plots created through EIGENSOFT where the first three principal components are plotted. Figures a and b use the Kidd55 SNP panel (K = 3), c and d use the Setser80 (K = 4), and e and f use the Seldin128 (K = 3). Abbreviations used: HUR = Honduras, DOM = Dominican Republic, COL = Colombia, CUB = Cuba, PUR = Puerto Rico, PCA = principal components analysis.

We evaluated the ability of a newly developed Hispanic AIMs panel (the Setser80) versus the Seldin128^9^ and Kidd55^11^ to separate heterogeneous Hispanic populations in the GOAL dataset (from Moreno-Estrada *et al*.^25^) using STRUCTURE^26^ and principal components analysis (PCA). With the STRUCTURE^26^ results, we applied the Evanno method^27^ which optimized the computer-determined (K) populations; the highest likelihood for the Setser80 was at K = 4 while Seldin128^9^ and Kidd55^11^ were optimized at K = 3 (Fig. 1a,1c,1e). The genetic proportions from STRUCTURE^26^ indicated that the Setser80 clearly separates HUR (Cluster 1 = 0.8290), DOM (Cluster 2 = 0.6976), and COL (Cluster 3 = 0.6562) (Table 1); but CUB (Cluster 2 = 0.2892, Cluster 4 = 0.6125) and PUR (Cluster 2 = 0.2048, Cluster 4 = 0.4145) remain indistinguishable (Fig. 1c). Using the genetic proportions from STRUCTURE^26^ for the Seldin128^9^ and Kidd55^11^ panels, HUR and COL separated predominately into Cluster 1 (HUR: Seldin128 = 0.7274, Kidd55 = 0.7258)(COL: Seldin128 = 0.5370, Kidd55 = 0.5311) (Table 1), but the remaining populations did not separate into distinct clusters.

**Table 1:** Genetic proportions from STRUCTURE.

Panel	Population	Cluster 1	Cluster 2	Cluster 3	Cluster 4	Individuals
Setser80 (K = 4)	HUR	0.8290	0.0387	0.0647	0.0676	13
Setser80 (K = 4)	DOM	0.0811	0.6976	0.1147	0.1067	21
Setser80 (K = 4)	COL	0.1601	0.0474	0.6562	0.1365	53
Setser80 (K = 4)	CUB	0.0348	0.2892	0.0634	0.6125	55
Setser80 (K = 4)	PUR	0.0836	0.2048	0.2969	0.4145	18
Seldin128 (K = 3)	HUR	0.7274	0.1155	0.1570	N/A	13
Seldin128 (K = 3)	DOM	0.2296	0.4283	0.3422	N/A	21
Seldin128 (K = 3)	COL	0.5370	0.1280	0.3349	N/A	53
Seldin128 (K = 3)	CUB	0.1672	0.3507	0.4822	N/A	55
Seldin128 (K = 3)	PUR	0.3415	0.2728	0.3860	N/A	18
Kidd55 (K = 3)	HUR	0.7258	0.1077	0.1664	N/A	13
Kidd55 (K = 3)	DOM	0.2664	0.3548	0.3788	N/A	21
Kidd55 (K = 3)	COL	0.5311	0.0690	0.4001	N/A	53
Kidd55 (K = 3)	CUB	0.1723	0.2528	0.5749	N/A	55
Kidd55 (K = 3)	PUR	0.3907	0.1705	0.4389	N/A	18

Each vertical line in a STRUCTURE diagram represents one individual, and the values listed here correspond to the genetic proportions of each of “K” computer determined populations, represented as colors in the diagram. The Setser80 categorized genetic proportions of samples into four computer-determined populations (K = 4). The Seldin128 and Kidd55 categorized genetic proportions into three computer-determined populations (K = 3).

We performed a principal components analysis (PCA) for the AIMs panels in the GOAL population (Fig. 1b,1d,1f). In the PCA of the Setser80, HUR clearly separated across PC1 and PC2, DOM separated from HUR across PC2, and COL separated from HUR across PC1 and from DOM across PC1 and PC2, which occupies three separate quadrants of the PCA (Fig. 1d). Seldin128^9^ PCA showed HUR and COL separated together but apart from the other populations across PC1, and CUB and DOM separated together along PC2 (Fig. 1f). The Kidd55^11^ performed poorly in PCA (Fig. 1b), not forming recognizable clusters, consistent with the genetic proportions generated in STRUCTURE^26^ (Fig. 1a) (Table 1). The Setser80 was able to differentiate HUR, DOM, and COL by the two different algorithms underlying STRUCTURE^26^ and PCA.

### Classification of unknowns

Based on the GOAL^25^ and 1000 Genomes Project^28^ (1000 G) allele frequencies, we modeled populations to determine classification accuracy using the Snipper 2.5 app suite^29^. Snipper uses naïve Bayesian likelihood ratios and multinomial logistic regression (MLR) for prediction of unknowns via −log(likelihood)^29^. Despite the different algorithms, both analyses had similar results.

As expected, the Setser80 had the highest overall accuracy across the three panels in the simulated GOAL dataset (98.4%) by naïve Bayesian classification implemented via leave-one-out cross-validation. Additionally, the Setser80 achieved 90% accuracy in the 1000 G dataset and 91.5% in the 7 Populations Combined dataset, both of which include populations not involved in our SNP ascertainment (Table 2). In the latter, the Setser80 panel (98%) and the Seldin panel (98.8%) achieved approximately equal accuracy in PEL, a population on which the Setser80 was not trained. In the 1000 G simulations, the Seldin panel was more accurate overall (92.4%) in comparison to the Setser80 (90%).

**Table 2:** Naïve Bayesian classification accuracy.

SNP Panel	Dataset	HUR	DOM	COL	CUB	PUR	PEL	MXL	Overall
Setser80	GOAL	100% (±0%)	96.8% (±2.5%)	99.4% (±0.5%)	96.8% (±2.8%)	99% (±0.7%)	N/A	N/A	98.4%
Seldin96	GOAL	99.2% (±0.4%)	89.6% (±3%)	78.4% (±4.2%)	76% (±3.3%)	90.8% (±1.8%)	N/A	N/A	87.9%
Kidd44	GOAL	88.4% (±3.4%)	78.6% (±4.1%)	67.6% (±4%)	66.2% (±5.3%)	68% (±7.3%)	N/A	N/A	73.8%
Setser80	1000 G	N/A	N/A	81.9% (±2.7%)	N/A	90.4% (±2.1%)	98.1% (±0.9%)	89.8% (±3%)	90%
Seldin96	1000 G	N/A	N/A	84.2% (±3.6%)	N/A	89.8% (±4.9%)	99.4% (±0.7%)	96.3% (±1.5%)	92.4%
Kidd44	1000 G	N/A	N/A	63.2% (±1.9%)	N/A	75.84% (±3%)	91.84% (±2.7%)	85.28% (±3.3%)	79.00%
Setser80	7 Pops	98.4% (±0.9%)	97.4% (±1.7%)	77.6% (±8.2%)	95.8% (±1.9%)	89.8% (±2.9%)	98% (±1%)	83.4% (±3.3%)	91.5%
Seldin96	7 Pops	85% (±2.5%)	84.4% (±3.1%)	79.8% (±4.6%)	68.8% (±3.1%)	79.6% (±7%)	98.8% (±0.8%)	96.2% (±0.8%)	84.7%
Kidd44	7 Pops	67.8% (±7.8%)	83.2% (±5.1%)	59% (±4.4%)	61.2% (±4.3%)	56.4% (±2.1%)	91.4% (±1.1%)	78.6% (±4.6%)	71.1%

Comparison of the nine possible combinations of each of three simulated datasets on each of three SNP panels and their naïve Bayesian classification accuracy for each population. Reported as percent accuracy with two-tailed standard deviations listed in parentheses (). Abbreviations used: GOAL = Genomic Origins and Admixture in Latinos, 1000 G = 1000 Genomes Project, 7 Pops = 7 Populations Combined, COL = Colombia, CUB = Cuba, DOM = Dominican Republic, HUR = Honduras, PUR = Puerto Rico, PEL = Peru from Lima, and MXL = Mexicans living in Los Angeles. Both Colombian populations from GOAL and 1000 G are listed in this table as “COL”.

Naïve Bayes analysis of the actual 1000 G genotypes revealed the Setser80 had the highest specificity in CLM (98.4%), the highest sensitivity in MXL (84.4%), and similar specificity in PUR (85.2%) and PEL (97.7%) in comparison to the Seldin (86.8%, 95.4%) and Kidd (85.2%, 94.7%) panels (Table 3). In all three SNP panels, the micro-simulations underestimated the positive predictive value of CLM. The positive predictive value of Setser80 for PUR (simulated = 69.8%, real = 70.2%) and PEL (simulated = 91.8%, real = 89.8%) was concordant between the simulated and real data where it was either under or overestimated by the Seldin and Kidd panels. Both the Setser80 (simulated = 59.3%, real = 36.7%) and the Seldin (simulated = 80.1%, real 54.1%) panels overestimated positive predictive value in MXL while the Kidd panel values were concordant between the simulations (47.3%) and real genotypes (45.7%).

**Table 3:** Positive predictive values from naïve Bayes analysis.

Known Origin	SNP Panel	5 sets of 500 micro-simulations			347 real 1000 G genotypes		
		Sen. (%)	Spe. (%)	PPV (%)	Sen. (%)	Spe. (%)	PPV (%)
CLM	Setser80	81.9%	70.1%	47.8%	17.0%	98.4%	80.0%
	Seldin96	84.2%	77.9%	55.9%	55.3%	90.9%	69.3%
	Kidd44	63.2%	49.9%	29.6%	51.1%	83.8%	53.9%
PUR	Setser80	90.4%	86.9%	69.8%	81.7%	85.2%	70.2%
	Seldin96	89.8%	80.5%	60.6%	89.4%	86.8%	74.4%
	Kidd44	75.8%	51.7%	34.4%	71.2%	85.2%	67.3%
PEL	Setser80	98.1%	97.1%	91.8%	62.4%	97.7%	89.8%
	Seldin96	99.4%	98.9%	96.9%	87.1%	95.4%	86.0%
	Kidd44	91.8%	90.4%	76.1%	75.3%	94.7%	82.1%
MXL	Setser80	89.8%	79.5%	59.3%	84.4%	67.1%	36.7%
	Seldin96	96.3%	92.0%	80.1%	51.6%	90.1%	54.1%
	Kidd44	85.3%	68.3%	47.3%	50.0%	86.6%	45.7%

Sensitivity, specificity, and positive predictive values from naïve Bayes leave-one-out cross-validation for the average of five sets of 500 micro-simulations (left) and n = 347 actual 1000 G genotypes (right). Micro-simulations were generated based on the allele frequencies from the 1000 G dataset only. Abbreviations used: Sen. = sensitivity, Spe. = specificity, PPV = positive predictive value, CLM = Colombia from Medellin, PUR = Puerto Rico, PEL = Peru from Lima, and MXL = Mexicans living in Los Angeles.

Utilizing the MLR algorithm, Setser80 had the highest accuracy in GOAL and 7 Populations Combined (99% and 93.2%, respectively); the Setser80 and Seldin panel had equal accuracy in 1000 G (93.8%); and the Kidd panel had 80.5% in GOAL, 71.4% in 7 Populations Combined, and 82.2% overall in 1000 G (Table 4). As expected, HUR achieved >95% accuracy in the Setser80 and the Seldin panel across all datasets. Surprisingly, PEL also achieved >95% and MXL > 90% accuracies using the Setser80, although the Setser80 had not been trained on these populations.

**Table 4:** MLR classification accuracy.

SNP Panel	Dataset	HUR	DOM	COL	CUB	PUR	PEL	MXL	Overall
Setser80	GOAL	100% (±0%)	100% (±0%)	100% (±0%)	97.5% (±5%)	97.5% (±5%)	N/A	N/A	99%
Seldin96	GOAL	97.5% (±5%)	95% (±5.8%)	85% (±12.9%)	90% (±11.5%)	95% (±5.8%)	N/A	N/A	92.5%
Kidd44	GOAL	92.5% (±9.6%)	90% (±0%)	75% (±17.3%)	72.5% (±15%)	72.5% (±9.6%)	N/A	N/A	80.5%
Setser80	1000 G	N/A	N/A	90.4% (±7.4%)	N/A	90.4% (±7.4%)	100% (±0%)	94.2% (±7.4%)	93.8%
Seldin96	1000 G	N/A	N/A	94.2% (±3.8%)	N/A	88.5% (±7.7%)	100% (±0%)	92.3% (±6.3%)	93.8%
Kidd44	1000 G	N/A	N/A	76.9% (±8.9%)	N/A	76.9% (±6.3%)	92.3% (±8.9%)	82.7% (±9.7%)	82.2%
Setser80	7 Pops	95% (±5.8%)	97.5% (±5%)	77.5% (±9.6%)	100% (±0%)	92.5% (±9.6%)	100% (±0%)	90% (±8.2%)	93.2%
Seldin96	7 Pops	100% (±0%)	82.5% (±20.6%)	82.5% (±12.6%)	85% (±17.3%)	67.5% (±17.1%)	97.5% (±5%)	100% (±0%)	87.9%
Kidd44	7 Pops	57.5% (±9.6%)	85% (±12.9%)	55% (±5.8%)	72.5% (±12.6%)	55% (±12.9%)	92.5% (±9.6%)	82.5% (±9.6%)	71.4%

Comparison of the nine possible combinations of each of three simulated datasets on each of three SNP panels and their MLR classification accuracy for each population. Reported as percent accuracy with two-tailed standard deviations listed in parentheses (). Abbreviations used: GOAL = Genomic Origins and Admixture in Latinos, 1000 G = 1000 Genomes Project, 7 Pops = 7 Populations Combined, COL = Colombia, CUB = Cuba, DOM = Dominican Republic, HUR = Honduras, PUR = Puerto Rico, PEL = Peru from Lima, MXL = Mexicans living in Los Angeles, and MLR = multinomial logistic regression. Both Colombian populations from GOAL and 1000 G are listed in this table as “COL”.

Despite performing best overall, the Setser80 did misclassify COL 22.5% of the time in the 7 Populations Combined dataset (Supplemental Table S3). When it misclassified COL, the individual was classified as MXL 77.8% and PUR 22.2% of the time. Conversely, even though MXL classified correctly 90% of the time, when individuals were misclassified they were misclassified as COL 100% of the time. In comparison, the Seldin panel misclassified COL 17.5% of the time spread across four countries, primarily into PUR (10%). The Kidd panel exhibited a similar trend where COL misclassified into five countries: PUR (15%), MXL (10%), HUR (7.5%), CUB (7.5%), and DOM (2.5%) in addition to one individual which could not be classified. When MXL was misclassified using the Kidd panel, it misclassified into PEL (7.5%), HUR (5%), and COL (5%). Additionally, the Kidd panel had high misclassification of HUR into MXL (20%), COL (15%), and PUR (7.5%).

## Discussion

We report a panel of 80 AIMs for Hispanic BGA classification using Weir & Cockerham’s estimator^30^ of Wright’s F_ST_^31^. Honduras (HUR) and DOM emerged first in STRUCTURE^26^ and PCA, followed by COL at K = 4, which separated from CUB & PUR, indicating three distinct populations (Table 1). Based on the allele frequencies, we created a series of micro-simulations to compare the BGA classification of the Setser, Seldin, and Kidd panels. Overall, the Setser80 outperformed the Seldin and Kidd panels in naïve Bayesian classification and MLR classification accuracies in the GOAL dataset (naïve Bayes = 98.4%, MLR = 99%) and the 7 Populations Combined (naïve Bayes = 91.5%, MLR = 93.2%). Notably, PEL and MXL were classified with >95% and >80% accuracy, respectively, indicating the Setser80 panel is portable into other Hispanic datasets and populations.

Many panels have sought country-level ancestry determination, using a variety of SNP ascertainment methods^19,21,28,29^. Continentally, the EUROFORGEN Global AIMs^12^ and the Kidd55^11^ panel used allele frequency differentials (δ). Within a country, the United States HapMap 3 populations^20^ used PCA with receiver operating characteristics curve (ROC)^19^, and the Mexican mestizos panel used nested subsets with high SNP weights followed by the lowest number of SNPs with the highest PC1^21^. Similar to Kidd *et al*.^11^, we prioritized SNPs that distinguished populations with lower mean F_ST_ per country. However, we focused on differentiating Hispanic instead of continental populations. Kosoy *et al*.^9^ (Seldin128) also concentrates on continental differentiation, but they also evaluated their AIMs on African American, Puerto Rican, and Mexican/Mexican American populations.

We used the Snipper 2.5 app suite^29^ that provided two classification methods: a naive Bayesian classifier and MLR^32^. This web-based classifier was designed for classification of externally visible characteristics^33–37^ and ancestry^12,38–41^, particularly in forensics. Snipper has successfully analyzed admixed South American populations^34,42,43^, similar to those used here.

The classification accuracy of the Seldin and Kidd panels is due to both the composition of their SNP ascertainment datasets and the size of the panels. The Seldin panel (96.2%, 96.3%) was more accurate in MXL than the Setser80 (83.4%, 89.8%) in the 7 Populations Combined and 1000 G datasets, respectively. Its success is likely because 199 of their 825 samples were from admixed Latin American and Amerindian individuals (Mexico and Puerto Rico especially)^9^. The Kidd panel emphasized capturing diversity by using 63 global populations^11^ including seven isolated Amerindian populations; they continue to add more populations via ALFRED^44^. The size of the Kidd panel and the ratio of SNPs to the number of samples (Kidd55 = 55 SNPs / 3071 samples = 0.0179; Seldin128 = 128 SNPs / 825 samples = 0.1552) suggests the number of SNPs, rather than SNP ascertainment population size, is the higher contributing factor to population differentiation. However, the number of individuals per population may also be a factor.

Our study’s limitations include: genechip design, sample size and its effect on allele frequencies. The GOAL^25^ study genechip^45^ was built on 270 African (YRI), Caucasian (CEU), and East Asian population (CHB and JPT) samples from HapMap 1^46^, without any Amerindian component. Although, our SNP ascertainment dataset was small it was not inconsistent with other studies^11,18,20^ where the larger overall size was coupled with small sub-populations. Therefore, we combined the GOAL^25^ dataset with the 1000 Genomes Admixed American dataset (n = 347)^28^, merging COL with CLM (n = 147) and PUR with PUR (n = 122) due to negligible allele frequency differences, to create the 7 Populations Combined.

The design of the Setser80 is based on the balance of the countries via country attributable mean F_ST_ and selection of SNPs with LD < 0.7. Using a dilution series of 234 to 44 SNPs, we evaluated the effect of panel size on classification accuracy in relation to Seldin and Kidd sized panels and found 80 SNPs to be sufficient. Therefore we chose 80 SNPs from 247 candidates by selecting SNPs such that ~20% could be attributed to each country. It is possible that other panels informative of Hispanic ancestry could be selected from the same candidates, but testing multiple different panels was beyond the scope of this study. Residual LD is possible despite our threshold where four pairs of SNPs had r^2^ > 0.5; however, removing one of each pair and classifying two separate 76 SNP subsets had negligible effect on classification accuracy via naïve Bayes (Supplemental Table S4) or MLR (Supplemental Table S5). By treating these loci as independent, we may underestimate accuracy as Kidd *et al*. 2013 has shown that diplotypes are effective predictors of ancestry^47^.

We used micro-simulations in this study in order to normalize the size of each population and expand the analysis to seven Hispanic populations instead of the four publicly available through the 1000 Genomes Project^28^. Although real genotypes would have been preferable, widely variable population sizes could disproportionately affect the classification accuracy for smaller populations, as may have been the case with the real MXL genotypes. Our analysis of additional populations is a more realistic representation of the challenges of a more granular classification of heterogeneous populations. Forensic labs may not have access to a sizeable Hispanic database of individuals from multiple different countries; therefore, we simulated datasets based on readily available allele frequencies from multiple sources. By doing so, we have allowed MXL to misclassify into HUR which otherwise do not exist within the same dataset.

Additionally, our use of a static model for BGA determination may have overestimated classification success; despite reasonable success by other research groups^48^. Finally, our imputation of the Seldin128^9^ and Kidd55^11^ into the GOAL^25^ dataset required removal of ~30 loci to comply with the Setser80 QC filters. Missingness was not detrimental here because STRUCTURE disregards it^49,50^, and at 10% MLR is robust^49^. Alternatively, some missingness in micro-simulations may approximate the degraded forensic samples^51^.

Our findings indicate that the Setser80 can predict BGA of individuals of presumed Hispanic origin with high confidence. By selecting additional SNPs attributed to countries with lower average country attributable F_ST_ (COL, CUB, and PUR) to create the panel, the Setser80 had similar accuracy overall in GOAL^25^ and 7 Populations Combined. The Setser80 is robust as it clusters well with Bayesian model-based clustering and PCA, and classifies equally well in naïve Bayes classification and MLR. The Setser80 is portable and, to our knowledge, is the first BGA AIMs panel specifically for the Caribbean and surrounding mainland countries. In comparison to Seldin128^9^, Kidd55^11^, and 46 Consensus SNPs^24^, our 80 AIMs for Hispanic BGA is unique, both exact and by linkage disequilibrium. Therefore, it is our intention that the Setser80 be integrated into a future Western Hemisphere panel.

## Materials and Methods

### Genomic Origins and Admixture in Latinos (GOAL) dataset

Here we downloaded the GOAL dataset and used 160 unrelated individuals including Honduran (HUR, n = 13), Dominican Republican (DOM, n = 21), Colombian (COL, n = 53), Cuban (CUB, n = 55), and Puerto Rican (PUR, n = 18) populations with three of four grandparents from the same country^25^. These samples were collected in South Florida and genotyped using the Affymetrix 6.0 gene chip of 906,600 predetermined SNPs^45^.

The Genomic Origins and Admixture in Latinos (GOAL) dataset analyzed during the current study is available in the dbGaP repository, accession number phs000750.v1.p1, found at: https://www.ncbi.nlm.nih.gov/projects/gap/cgi-bin/study.cgi?study_id=phs000750.v1.p1&phv=202273&phd=4443&pha = &pht=3936&phvf = &phdf = &phaf = &phtf = &dssp=1&consent = &temp=1. Funding support for the GOAL Study was provided by the National Institute of General Medical Sciences (1R01GM090087). Additional support for sample collection was provided by a grant from the Stanley J. Glaser Foundation and the Dr. John T. Macdonald Foundation Department of Human Genetics.

### Genomes (1000 G) dataset

For further comparison, we used fully sequenced individuals from the 1000 Genomes Project Phase 3 Admixed American populations (n = 347)^28^, accessed through the UCSC Genome Browser^52^. These include Colombia in Medellin (CLM, n = 94), Peru in Lima (PEL, n = 85), Puerto Rico (PUR, n = 104), and Mexican Living in Los Angeles (MXL, n = 64)^28^. The 1000 Genomes Project dataset is available via the UCSC Genome Browser, found at: http://genome.ucsc.edu/.

### SNP ascertainment

We created our AIMs panel by applying a series of quality control algorithms. Beginning with 897,336 autosomal SNPs on the genechip^45^, we filtered the GOAL dataset by linkage disequilibrium (LD) ≤ 0.7, missingness ≤ 0.1, and minor allele frequency (maf) ≥ 0.01 using PLINK v.1.9^53,54^ and retained 494,886 SNPs. After calculating F_ST_^31^ by Weir & Cockerham’s algorithm^30^ in PLINK v.1.9 (https://www.cog-genomics.org/plink/1.9/basic_stats#fst)^55^, 1509 SNPs with F_ST_ ≥ 0.15 for at least one pairwise comparison were retained.

We calculated the mean F_ST_ for each of the five countries and assigned each SNP to a country based on the highest mean F_ST_. The next highest mean F_ST_ was designated the 2^nd^ country mean F_ST_. For example, rs3777908 is attributed to HUR because the average of HUR vs. DOM, HUR vs. COL, HUR vs. CUB, and HUR vs. PUR is [(0.27318 + 0.19754 + 0.19560 + 0.28808)/4] = 0.23860, which was the highest country mean F_ST_ value for rs3777908. The 2^nd^ highest country mean F_ST_ = 0.07442, corresponded to PUR (see Supplemental Table S1 for example calculations).

We binned the 1509 SNPs by the 1^st^ and 2^nd^ highest country attributable mean F_ST_ and removed SNPs where the 1^st^ country mean F_ST_ < 0.11 and 2^nd^ country mean F_ST_ < 0.09, resulting in 437 SNPs. Since 63.3% of the 1509 candidate SNPs were attributable to HUR or DOM, we removed SNPs where HUR and DOM had the 1^st^ and 2^nd^ highest country mean F_ST_, where HUR had the 2^nd^ highest country mean F_ST_, and the 100 lowest ranked SNPs where HUR or DOM had the highest country mean F_ST_. From the remaining 247 SNPs, we chose a subset of 80 in order to maintain ~20% contribution of SNPs for each country across 1^st^ and 2^nd^ country attribution. Therefore, we proceeded with the Setser80 (Supplemental Table S2), which has the following country attributable mean F_ST_ values: HUR (mean F_ST_ = 0.21228), DOM (mean F_ST_ = 0.16901), COL (mean F_ST_ = 0.14212), CUB (mean F_ST_ = 0.10803), and PUR (mean F_ST_ = 0.10272).

To assess the value of our panel, we compared it to two commonly sited AIMs panels^9,11^. Here, we refer to the panel developed by Kosoy *et al*., 2009 as the Seldin128^9^, and the 55 ancestry informative SNPs developed by Kidd *et al*., 2014 as the Kidd55^11^. We performed each analysis on the Setser80 in parallel with the Kidd and Seldin panels to evaluate the utility of our Hispanic AIMs panel.

### Imputation

The SNPs on the Affymetrix 6.0 gene chip^45^ were pre-determined and not all SNPs were included in the ABI Taqman assay used to genotype the Seldin128^9^ and Kidd55^11^; therefore, we imputed these two panels into the GOAL dataset^25^ using IMPUTE2^56^ on the full 250 individuals using a 5 Mb window centered on each SNP and an effective population size of 20,000 as seen in Instructions for IMPUTE version 2^57^. We used 2,504 individuals from 1000G^28^ for the genetic map and legend and the strand alignment from dbSNP batch query. Given the use of genome builds NCBI35/hg17 to GRCh38/hg38, we converted all components to GRCh37/hg19 for analysis.

However, the gene chip used^45^ was based on an early genome build (NCBI35/hg17) which did not have all the tag SNPs necessary (in comparison to the 1000 G Project) to reliably impute ~30 of the SNPs from Seldin128^9^ and 11 from Kidd55^11^ for each individual. We assessed the accuracy of the imputation using the concordance tables provided by IMPUTE2; of the ~160 imputed SNPs from 20 chromosomes the mean concordance = 92.6% and range = 85.3% to 96.4%. Of the ~30 SNPs with missingness >10%, there was no obvious pattern between missingness proportion and concordance. Despite multiple attempts with different intervals, rs10954737 from the Seldin128^9^ was unable to be imputed due to the lack of Panel 2 SNPs. Because STRUCTURE and PCA ignore missing data^49,50^, the full Seldin128^9^ and Kidd55^11^ were used in these analyses. However, since the resampling approach to simulations is dependent upon the reliability of allele frequencies in our real data^58^, we applied the same <10% missingness filter used in the development of the Setser80; this resulted in 96 SNPs in the Seldin panel and 44 SNPs in the Kidd panel after imputation.

### STRUCTURE

We evaluated ancestry by the Bayesian model-based clustering method used in STRUCTURE v.2.3.4^26^ to compare the self-reported to computer-determined (K) populations. We performed STRUCTURE analysis at K = 2 to K = 7 for each dataset/panel at 10 iterations each using the admixture model, no LOCPRIOR, 10,000 burn-in, and 100,000 Markov Chain Monte Carlo (MCMC) repetitions. The final STRUCTURE diagrams for each SNP panel were optimized and averaged through STRUCTURE Harvester^59^, CLUMPP^60^, and Distruct^61^ to create the diagrams in Fig. 1.

### Principal components analysis (PCA)

We analyzed the Setser80, Seldin128^9^, and Kidd55^11^ on the GOAL dataset by PCA using EIGENSOFT v.6.1.4^62^ and plotted the first three eigenvectors. Genesis^63^ was used for improved visualization of clustering as seen in Fig. 1.

### Linkage disequilibrium (LD) analysis

Using the web-based tool LDmatrix^64^, we compared the Setser80 to the Seldin128^9^ and Kidd55^11^, and the 46 Consensus SNPs described in a review article by Soundararajan *et al*.^24^. We used r^2^ > 0.7 as the threshold to evaluate whether any SNP in the Setser80 was in strong LD with SNP(s) from Seldin128^9^ and Kidd55^11^ (tested together) or the 46 Consensus SNPs appearing in more than 3 of 21 panels of AIMs^24^.

### Modeling for the prediction of unknowns

To model the data for BGA prediction of unknown individuals, we used a resampling approach based on calculated allele frequencies of the three SNP panels on each dataset^58^. We simulated a randomly mating population of 100–125 individuals within each country. Next, we assigned a genotype to individuals by generating a random number between 1 and 0 and comparing this number to the maf for the country at the specified locus. Any random number above the maf was assigned the major allele. All genotypes were created from 2 separate allele generations for each locus. The simulation of each population was performed at least 5 times for the GOAL and 1000 G countries. The 7 Populations Combined dataset was created by merging the countries from the 1000 G and GOAL simulations without regard to simulation number. We verified our model using a chi-square test for each panel and found the allele frequencies from the simulation sets were not significantly different from the true allele frequencies at α = 0.05 after Bonferroni correction.

### Classification of unknowns

Snipper 2.5 app suite^29^ is a web-based Naïve Bayes classifier, found here (http://mathgene.usc.es/snipper/), which calculates −log(likelihood) with leave-one-out cross-validation and multinomial logistic regression (MLR) options. Cross-validation divides a set of data into a training set and a testing set, and rotates the samples until all samples have been in the testing set. Using the “Thorough analysis of population data with a custom Excel file” option, Snipper calculated likelihood ratios (LR) of *population vs. not the population* and selected the country that corresponded to the highest LR. MLR is similar to STRUCTURE^26,32^, which calculated genetic proportions of individuals (as percent admixture) instead of whole populations, and categorized individuals based on those probabilities. We used 100–125 micro-simulations (individuals) each from population for references and selected 10% of profiles from a separate set of micro-simulations to predict unknowns. We evaluated potential overlap of MLR classification using the confusion matrix and assessed the validity of our classification by sensitivity, specificity, and positive predictive value from the naïve Bayes classification of the actual 1000 G genotypes (n = 347; CLM = 94, PUR = 104, PEL = 85, and MXL = 64).

### Ethical approval and informed consent

This research study using the Genomic Origins and Admixture in Latinos (GOAL) from Moreno-Estrada, A. *et al*. (2013)^25^, and the 1000 Genomes Project^28^ datasets was approved under University of North Texas Health Science IRB 2013-201. As this manuscript only used pre-existing genetic data from Moreno-Estrada, A. *et al*. (2013)^25^, where their “Informed consent was obtained from all participants under approval by the University of Miami Institutional Review Board (study no. 20081175)”. The 1000 Genomes Project data was only included in the International Genome Sample Resource if the submission was in accordance with the Consent, Ethics Review and Sampling Process of the 1000 Genomes Project^28^.

## Supplementary information

Supplementary Information.
