# Supplementary material for: Differentiation of Hispanic biogeographic ancestry with 80 ancestry informative markers
Source: Sci Rep. 2020 May 8;10:7745. doi: 10.1038/s41598-020-64245-4 (PMC7210943; doi:10.1038/s41598-020-64245-4)
Supplement: Supplementary file 1 — Supplementary Information. [file 41598_2020_64245_MOESM1_ESM.pdf]

Supplementary Information for:

Differentiation of Hispanic biogeographic ancestry with 80 ancestry informative markers

Casandra H. Setser[1], John V. Planz[1], Robert C. Barber[1], Nicole R. Phillips[1], Ranajit Chakraborty[1, 2] & Deanna S. Cross[1]
[1] University of North Texas Health Science Center; Department of Microbiology, Immunology, and Genetics
[2] Deceased

Supplemental Table S1: Country attributable mean F_{ST} calculations

CHR	SNP	COL vs. CUB F_{ST}	COL vs. DOM F_{ST}	COL vs. HUR F_{ST}	COL vs. PUR F_{ST}	CUB vs. DOM F_{ST}	CUB vs. HUR F_{ST}	CUB vs. PUR F_{ST}	DOM vs. HUR F_{ST}	DOM vs. PUR F_{ST}	HUR vs. PUR F_{ST}
6	**rs3777908**	0.01129	0.00482	**0.19754**	0.00327	0.00583	**0.19560**	0.00425	**0.27318**	0.00207	**0.28808**
3	**rs9857908**	**0.12265**	**0.15319**	**0.21601**	**0.15047**	0.20331	0.10969	0.07975	0.15533	0.09551	0.07984
5	**rs871234**	0.07459	**0.17146**	0.09206	0.05378	**0.15663**	0.01475	0.00150	**0.24140**	**0.24402**	0.03115
9	**rs10981894**	**0.09794**	0.07058	0.10767	0.13719	**0.18782**	**0.11750**	**0.12768**	0.07918	0.10264	0.13199
10	**rs16932385**	0.02925	0.01002	**0.26014**	0.00765	0.06678	**0.22826**	0.06055	**0.30385**	0.02558	**0.31814**
12	**rs2051827**	**0.13258**	**0.14135**	**0.21359**	**0.14807**	0.09160	0.09166	0.14127	0.03493	0.03900	0.07688
14	**rs178384**	**0.10567**	0.07545	0.13410	0.10048	**0.15544**	**0.11984**	**0.10358**	0.05628	0.02126	0.05578
16	**rs7198325**	0.09113	**0.19367**	0.03953	0.05931	**0.18518**	0.01100	0.02889	**0.27872**	**0.25715**	0.00333
21	**rs2834567**	**0.14717**	**0.15771**	**0.20497**	**0.16125**	0.11549	0.08042	0.10691	0.02358	0.03044	0.01768
21	**rs1000472**	**0.16861**	**0.18993**	**0.20436**	**0.19454**	0.08235	0.04620	0.05654	0.03668	0.04488	0.00699

CHR	SNP	Max F_{ST}	Mean F_{ST}	Min F_{ST}	COL F_{ST}	CUB F_{ST}	DOM F_{ST}	HUR F_{ST}	PUR F_{ST}	1st Country	2nd Country
6	**rs3777908**	0.09859	0.28808	0.00021	0.05423	0.05424	0.07148	**0.23860**	*0.07442*	HUR	PUR
3	**rs9857908**	0.13657	0.21601	0.00797	**0.16058**	0.12885	*0.15183*	0.14021	0.10139	COL	DOM
5	**rs871234**	0.10813	0.24402	0.00015	*0.09797*	0.06186	**0.20338**	0.09484	0.08261	DOM	COL
9	**rs10981894**	0.11602	0.18782	0.00706	0.10335	**0.13274**	0.11006	0.10909	*0.12488*	CUB	PUR
10	**rs16932385**	0.13102	0.31814	0.00076	0.07676	0.09621	0.10156	**0.27760**	*0.10298*	HUR	PUR
12	**rs2051827**	0.11109	0.21359	0.00349	**0.15890**	*0.11428*	0.07672	0.10426	0.10131	COL	CUB
14	**rs178384**	0.09279	0.15544	0.00213	*0.10392*	**0.12113**	0.07711	0.09150	0.07028	CUB	COL
16	**rs7198325**	0.11479	0.27872	0.00033	*0.09591*	0.07905	**0.22868**	0.08314	0.08717	DOM	COL
21	**rs2834567**	0.10456	0.20497	0.00177	**0.16778**	*0.11250*	0.08180	0.08166	0.07907	COL	CUB
21	**rs1000472**	0.10311	0.20436	0.00070	**0.18936**	0.08843	*0.08846*	0.07356	0.07574	COL	DOM

Supplemental Table S2: Description of Setser80 panel

CHR	SNP	POS (hg18)	1st Country F_{ST}	1st Country	2nd Country F_{ST}	2nd Country
1	rs12130873	14629827	0.27834	HUR	0.08626	PUR
1	rs6694897	25990886	0.16554	COL	0.11347	CUB
1	rs1570099	35071891	0.14964	COL	0.09674	CUB
1	rs10493701	81919788	0.21506	HUR	0.09218	CUB
1	rs1040424	208201005	0.15556	HUR	0.09197	CUB
2	rs10495889	41547938	0.16222	HUR	0.09780	CUB
2	rs17018313	80045074	0.23693	HUR	0.07268	PUR
2	rs7568419	177078765	0.22077	HUR	0.09977	PUR
2	rs11693873	197850455	0.15274	HUR	0.09289	CUB
2	rs4433950	239329179	0.23298	HUR	0.07530	PUR
3	rs9853146	12274313	0.14598	COL	0.14106	CUB
3	rs4685443	17205888	0.13890	COL	0.07537	DOM
3	rs259425	22041732	0.16228	COL	0.10253	DOM
3	rs2356298	51509761	0.16715	HUR	0.11188	CUB
3	rs11719358	139267512	0.12963	COL	0.12198	DOM
3	rs9857908	139318436	0.16058	COL	0.15183	DOM
3	rs1586861	140541186	0.16976	COL	0.14602	CUB
4	rs3910480	161880988	0.14157	COL	0.07363	CUB
5	rs1366363	29985377	0.20338	HUR	0.11796	CUB
5	rs3733838	42757753	0.21079	HUR	0.08908	CUB
5	rs1438745	85249943	0.22267	DOM	0.09995	PUR
5	rs16902270	85859474	0.16480	COL	0.10138	CUB
5	rs871234	174418204	0.20338	DOM	0.09797	COL
5	rs692713	176186041	0.20640	DOM	0.11848	COL
5	rs4608884	178979749	0.14133	COL	0.10817	CUB
5	rs190592	179263370	0.20994	DOM	0.13208	COL
6	rs6596807	1267033	0.20170	HUR	0.09021	CUB
6	rs9392285	1310265	0.19772	HUR	0.11633	CUB
6	rs9501948	3156786	0.17955	DOM	0.11352	COL
6	rs1329521	47840137	0.16820	COL	0.13484	CUB
6	rs17745021	73769618	0.22153	HUR	0.08811	PUR
6	rs3777908	111980345	0.23860	HUR	0.07442	PUR
6	rs17087570	156949439	0.25097	HUR	0.11388	CUB
6	rs4709836	164636380	0.14426	COL	0.10892	DOM
7	rs12536738	95370320	0.18567	HUR	0.11446	CUB
7	rs10953750	113424187	0.15649	HUR	0.09920	CUB
7	rs2352479	137589866	0.12823	COL	0.09400	DOM
7	rs17480133	145414794	0.19653	COL	0.11315	CUB
8	rs766382	60170978	0.11474	CUB	0.11356	COL

8	rs1588459	142143028	0.17997	COL	0.10768	CUB
9	rs6474712	12405123	0.23846	DOM	0.08651	COL
9	rs880397	12451349	0.22029	DOM	0.08143	COL
9	rs10981894	115508956	0.13274	CUB	0.12488	PUR
10	rs2008617	34499344	0.28372	DOM	0.10056	PUR
10	rs16912280	59880729	0.26838	DOM	0.10208	COL
10	rs1259603	76814933	0.23429	HUR	0.10085	PUR
10	rs16932385	77017391	0.27760	HUR	0.10298	PUR
10	rs11189628	100230671	0.15679	COL	0.10420	CUB
10	rs17112705	101927184	0.17958	COL	0.11240	CUB
11	rs1849352	61578905	0.18204	HUR	0.08943	CUB
11	rs2878712	132343165	0.22463	HUR	0.09477	PUR
12	rs10840730	17553352	0.22546	HUR	0.07914	CUB
12	rs2051827	46242298	0.15890	COL	0.11428	CUB
12	rs12146822	66857933	0.18173	HUR	0.15467	CUB
12	rs7310083	68347053	0.21863	PUR	0.13093	COL
12	rs1967232	115531283	0.14664	COL	0.10884	CUB
12	rs6486527	129231989	0.11961	COL	0.10008	DOM
13	rs9569702	56996265	0.15029	HUR	0.08848	CUB
13	rs4341647	77872864	0.17141	HUR	0.09511	CUB
13	rs9556940	97818960	0.16213	COL	0.10425	CUB
14	rs1957572	67806224	0.17737	HUR	0.09433	CUB
14	rs178384	79252594	0.12113	CUB	0.10392	COL
14	rs12434466	96394042	0.20049	HUR	0.10147	CUB
14	rs17094860	96560669	0.23314	HUR	0.10820	CUB
14	rs12435621	97182294	0.31859	HUR	0.09892	PUR
14	rs12431505	97188301	0.39018	HUR	0.12929	PUR
14	rs1462266	97319155	0.24835	HUR	0.09924	CUB
14	rs17097005	98013715	0.22045	HUR	0.08099	PUR
15	rs2869550	76768056	0.15132	HUR	0.09375	CUB
16	rs7198325	12572650	0.22868	DOM	0.09591	COL
16	rs4470161	78627029	0.20305	DOM	0.12023	COL
17	rs1019118	52103046	0.16663	COL	0.12606	DOM
17	rs17246021	67616034	0.18769	HUR	0.11189	CUB
17	rs12936629	67854083	0.22241	HUR	0.09781	PUR
20	rs221308	34709812	0.16671	COL	0.10029	DOM
20	rs6015771	58532301	0.16355	COL	0.14140	CUB
21	rs1013001	14581125	0.16833	HUR	0.10737	CUB
21	rs2834567	34950995	0.16778	COL	0.11250	CUB
21	rs440431	42905781	0.18410	DOM	0.12326	COL
21	rs1000472	43602306	0.18936	COL	0.08846	DOM

Supplemental Table S3: MLR confusion matrix

Known Origin	SNP Panel	HUR	DOM	COL	CUB	PUR	PEL	MXL	Total
HUR	Setser80	38	0	0	0	0	0	2	40
	Seldin96	40	0	0	0	0	0	0	40
	Kidd44	23	0	6	0	3	0	8	40
DOM	Setser80	0	39	0	1	0	0	0	40
	Seldin96	0	33	0	3	4	0	0	40
	Kidd44	1	34	0	4	1	0	0	40
COL	Setser80	0	0	31	0	2	0	7	40
	Seldin96	1	0	33	1	4	0	1	40
	Kidd44	3	1	22	3	6	0	4	39*
CUB	Setser80	0	0	0	40	0	0	0	40
	Seldin96	0	4	0	34	1	0	0	39*
	Kidd44	0	4	1	29	6	0	0	40
PUR	Setser80	0	0	0	3	37	0	0	40
	Seldin96	0	3	5	5	27	0	0	40
	Kidd44	2	3	8	4	22	0	1	40
PEL	Setser80	0	0	0	0	0	40	0	40
	Seldin96	0	0	0	0	0	39	1	40
	Kidd44	0	0	0	0	0	37	3	40
MXL	Setser80	0	0	4	0	0	0	36	40
	Seldin96	0	0	0	0	0	0	40	40
	Kidd44	2	0	2	0	0	3	33	40

Supplemental Table S4: Naïve Bayes classification of panels of 76 SNPs

SNP Panel	Dataset	COL	CUB	DOM	HUR	PUR	PEL	MXL	Overall
**76 SNPs (removed 1-3-5-7)**	**7 Pops**	75.6% (±6.2%)	95.2% (±2.5%)	97.4% (±1.5%)	98.2% (±0.8%)	89.2% (±5.0%)	97.4% (±1.3%)	84.2% (±2.3%)	91.0%
**Setser80**	**7 Pops**	77.6% (±8.2%)	95.8% (±1.9%)	97.4% (±1.7%)	98.4% (±0.9%)	89.8% (±2.9%)	98% (±1.0%)	83.4% (±3.3%)	91.5%
**76 SNPs (removed 2-4-6-8)**	**7 Pops**	78.6% (±5.2%)	95% (±1.6%)	96.6% (±1.1%)	97.8% (±0.8%)	88.8% (±3.3%)	98.4% (±0.5%)	82.6% (±2.9%)	91.1%

Supplemental Table S5: MLR classification of panels of 76 SNPs

SNP Panel	Dataset	COL	CUB	DOM	HUR	PUR	PEL	MXL	Overall
**76 SNPs (removed 1-3-5-7)**	**7 Pops**	**77.5% (±9.6%)**	**100% (±0.0%)**	**97.5% (±5.0%)**	**92.5% (±5.0%)**	**92.5% (±9.6%)**	**100% (±0.0%)**	**90% (±0.0%)**	**92.9%**
**Setser80**	**7 Pops**	**77.5% (±9.6%)**	**100% (±0.0%)**	**97.5% (±5.0%)**	**95% (±5.8%)**	**92.5% (±9.6%)**	**100% (±0.0%)**	**90% (±8.2%)**	**93.2%**
**76 SNPs (removed 2-4-6-8)**	**7 Pops**	**72.5% (±12.6%)**	**97.5% (±5.0%)**	**97.5% (±5.0%)**	**97.5% (±5.0%)**	**92.5% (±9.6%)**	**97.5% (±5.0%)**	**90% (±8.2%)**	**92.1%**

Supplemental Table S1: Country attributable mean F_{ST} calculations. This table gives examples of how the F_{ST} calculations are made for each of the five countries. Presented here are the ten pairwise F_{ST} comparisons for each SNP, the standard maximum/mean/minimum, the final country attributable mean F_{ST} values for each country (the mean of the four pairwise comparisons with one country in common in bold), and the names of the countries corresponding 1st and 2nd highest country attributable mean F_{ST} values. Abbreviations used: HUR = Honduras, DOM = Dominican Republic, COL = Colombia, CUB = Cuba, and PUR = Puerto Rico.

Supplemental Table S2: **Description of the Setser80 panel.** The Setser80 AIMs panel incorporates these 80 SNPs. Below appears the .map information (CHR = Chromosome, SNP = name of single nucleotide polymorphism, and POS = position in NCBI36/hg18 genome build). Mean F_{ST} lists the average F_{ST} across all 10 pairwise comparisons possible across 5 populations. The 1st country attributable mean F_{ST} and 2nd country attributable mean F_{ST} refers to the average of 4 pairwise comparisons that have one country in common. First country attributable mean F_{ST} refers to the largest, most divergent average and its corresponding population while the 2nd country attributable mean F_{ST} refers to the 2nd most divergent average and its population. Populations: HUR = Honduras, DOM = Dominican Republic, COL = Colombia, CUB = Cuba, and PUR = Puerto Rico.

Supplemental Table S3: MLR confusion matrix. Confusion matrix showing into which population(s) each known population classifies. This table reflects cumulative values from four sets of 70 micro-simulations (10 per population, per analysis) from the 7 Populations Combined dataset classified by MLR. Abbreviations used: COL = Colombia, CUB = Cuba, DOM = Dominican Republic, HUR = Honduras, PUR = Puerto Rico, PEL = Peru from Lima, and MXL = Mexicans living in Los Angeles. * One sample from this panel was unable to be classified for this population.

Supplemental Table S4: Naïve Bayes classification of panels of 76 SNPs. Evaluation of the effect of four pairs of SNPs with LD $r^2 > 0.5$ in the Setser80 on classification accuracy using the 7 Populations Combined dataset. One of each of the following pairs was removed for each subset of 76 SNPs where the number in parentheses corresponds to the SNPs removed: (1) rs11719358-rs9857908 (2), (3) rs6596807-rs9392285 (4), (5) rs1259603-rs16932385 (6), and (7) rs12435621-rs12431505 (8). Abbreviations used: 7 Pops = 7 Populations Combined, COL = Colombia, CUB = Cuba, DOM = Dominican Republic, HUR = Honduras, PUR = Puerto Rico, PEL = Peru from Lima, and MXL = Mexicans living in Los Angeles.

Supplemental Table S5: MLR classification of panels of 76 SNPs. Evaluation of the effect of four pairs of SNPs with LD $r^2 > 0.5$ in the Setser80 on classification accuracy using the 7 Populations Combined dataset. One of each of the following pairs was removed for each subset of 76 SNPs where the number in parentheses corresponds to the SNPs removed: (1) rs11719358-rs9857908 (2), (3) rs6596807-rs9392285 (4), (5) rs1259603-rs16932385 (6), and (7) rs12435621-rs12431505 (8). Abbreviations used: 7 Pops = 7 Populations Combined, COL = Colombia, CUB = Cuba, DOM = Dominican Republic, HUR = Honduras, PUR = Puerto Rico, PEL = Peru from Lima, and MXL = Mexicans living in Los Angeles.
